# Association between Food Insecurity and Procurement Methods among People Living with HIV in a High Resource Setting

**DOI:** 10.1371/journal.pone.0157630

**Published:** 2016-08-03

**Authors:** Aranka Anema, Sarah J. Fielden, Susan Shurgold, Erin Ding, Jennifer Messina, Jennifer E. Jones, Brian Chittock, Ken Monteith, Jason Globerman, Sean B. Rourke, Robert S. Hogg, Canada Food Security Study Team

**Affiliations:** 1 Department of Pediatrics, Harvard Medical School, Harvard University, Boston, Massachusetts, United States of America; 2 Division of Emergency Medicine, Department of Medicine, Boston Children's Hospital, Boston, Massachusetts, United States of America; 3 Division of International Nutrition, Department of Food, Nutrition and Health, Faculty of Land and Food Systems University of British Columbia, Vancouver, British Columbia, Canada; 4 School of Population and Public Health, University of British Columbia, Vancouver, British Columbia, Canada; 5 HIV/AIDS Drug Treatment Program, British Columbia Centre for Excellence in HIV/AIDS, St. Paul’s Hospital, Vancouver, British Columbia, Canada; 6 Immune Deficiency Clinic, St. Paul’s Hospital, Vancouver, British Columbia, Canada; 7 Pacific AIDS Network, Vancouver, British Columbia, Canada; 8 AIDS Vancouver, Vancouver, British Columbia, Canada; 9 Coalition des organismes communautaires québécois de lutte contre le sida, Montréal, Quebéc, Canada; 10 Ontario HIV Treatment Network, Toronto, Ontario, Canada; 11 Centre for Research on Inner City Health, Li Ka Shing Knowledge Institute, St. Michael’s Hospital, Toronto, Ontario, Canada; 12 Department of Psychiatry, University of Toronto, Toronto, Ontario, Canada; 13 Faculty of Health Sciences, Simon Fraser University; Burnaby, British Columbia, Canada; Public Health Agency of Canada, CANADA

## Abstract

**Objective:**

People living with HIV in high-resource settings suffer severe levels of food insecurity; however, limited evidence exists regarding dietary intake and sub-components that characterize food insecurity (i.e. food quantity, quality, safety or procurement) in this population. We examined the prevalence and characteristics of food insecurity among people living with HIV across British Columbia, Canada.

**Design:**

This cross-sectional analysis was conducted within a national community-based research initiative.

**Methods:**

Food security was measured using the Health Canada Household Food Security Scale Module. Logistic regression was used to determine key independent predictors of food insecurity, controlling for potential confounders.

**Results:**

Of 262 participants, 192 (73%) reported food insecurity. Sub-components associated with food insecurity in bivariate analysis included: < RDI consumption of protein (p = 0.046); being sick from spoiled/unsafe food in the past six months (*p =* 0.010); and procurement of food using non-traditional methods (p <0.05). In multivariable analyses, factors significantly associated with food insecurity included: procurement of food using non-traditional methods [AOR = 11.11, 95% CI: 4.79–25.68, p = <0.001]; younger age [AOR = 0.92, 95% CI: 0.86–0.96, p = <0.001]; unstable housing [AOR = 4.46, 95% CI: 1.15–17.36, p = 0.031]; household gross annual income [AOR = 4.49, 95% CI: 1.74–11.60, p = 0.002]; and symptoms of depression [AOR = 2.73, 95% CI: 1.25–5.96, p = 0.012].

**Conclusions:**

Food insecurity among people living with HIV in British Columbia is characterized by poor dietary quality and food procurement methods. Notably, participants who reported procuring in non-traditional manners were over 10 times more likely to be food insecure. These findings suggest a need for tailored food security and social support interventions in this setting.

## Introduction

Food security exists “when all people, at all times, have physical, social and economic access to sufficient, safe and nutritious food, which meets their dietary needs and food preferences for an active and healthy life” [1]. Implicit in this definition is the notion that people who are food insecure may consume insufficient, poor quality, or unsafe foods, and may procure food in legally or socially unacceptable ways [2]. Food insecurity is prevalent among people living with HIV in North America, and associated with numerous socio-demographic and behavioural factors including low-income status, unstable housing, and a history of tobacco and illicit drug use [3–5]. Additionally, evidence supports an association between food insecurity and symptoms of depression [3], greater acute care utilization [6] and adverse antiretroviral therapy (ART) outcomes, including suboptimal adherence[7], immunological and virological response [4, 8], and survival [9, 10].

In British Columbia, Canada, we found that 73% of people receiving ART were food insecure [3], suggesting a significant increase from sample estimates ten years ago [11]. Food insecurity among HIV-positive individuals in this setting has been independently associated with an increased risk of unprotected sex among HIV-positive injection drug users independent of HAART use [12] and a two-fold increased risk of mortality when controlling for potential socio-demographic and clinical confounders [9, 10]. Several nutritional, mental health and behavioural pathways have been hypothesized to link food insecurity with adverse HIV health outcomes, which may be amenable to intervention [13]. However a critical starting point to the development of appropriate clinical and public health responses is to delineate what sub-components of food insecurity (i.e. food insufficiency, poor dietary diversity, safety, or procurement methods) [2]characterize food access and nutrition among HIV-positive populations. We therefore sought to evaluate the relationship between food insecurity and its sub-components in a community-based sample of people living with HIV across the province of BC.

## Methods

### Community-Based Research (CBR)

Community-based research (CBR) enhances capacities and empowers communities by inviting their members’ equitable involvement as research partners. The main aim of CBR is to generate knowledge about health priorities with the broader goals of strengthening communities and improving quality of life by placing the issues and questions of community organizations and their clients at the forefront of the research agenda. The central tenet of CBR is the development of multidisciplinary, collaborative partnerships between community members and academic researchers to ensure that research is relevant, ethical and methodologically rigorous. Thus, the principles of CBR include a commitment to reciprocal capacity-building of community and academic partners, empowerment of communities through all stages of the research process, sharing of decision-making and information, action outcomes and social change [14, 15]. This national CBR study was led by the Ontario HIV Treatment Network (OHTN) in collaboration with 38 community-based AIDS service organizations (ASOs) and four universities across three of Canada’s most populated provinces: British Columbia (BC), Ontario (ON) and Quebec (QC). This study was funded by the Canadian Institutes of Health Research (CIHR) and specifically addressed a need for further evidence regarding the burden of food insecurity among people living with HIV in Canada (www.foodsecuritystudy.ca).

The study was guided by the Greater Involvement of People Living with HIV/AIDS (GIPA) Principle, understood by the Joint United Nations Programme on HIV/AIDS (UNAIDS) and Global Network of People Living with HIV/AIDS (GNP+) as a rights-based approach to the active and meaningful participation of people living with HIV in the design, implementation, and monitoring and evaluation of programs, policies and research [16, 17]. Factors associated with the effective implementation of GIPA in Canada include valuing lived experience, building trust, providing training and mentoring opportunities, ensuring financial compensation, and accommodating the unique needs of people living with HIV [18]. Aligned with the GIPA Principle, the national study involved a team of 33 HIV-positive peer research associates (PRAs) across Canada, including 10 in BC. PRAs were trained in interview techniques, self-care strategies and ethical conduct in research. PRAs were responsible for developing survey questions that they felt addressed the unique lived experience of people living with HIV; ensuring linguistic and cultural appropriateness of the overall quantitative survey tool; coordinating participant recruitment; leading voluntary informed consent process; administering interviews; recording survey results; facilitating community-based referral of participants to support services, as appropriate; and supporting knowledge translation and exchange activities.

### Participant Eligibility and Study Design

Participant eligibility for the national study included: being 19 years of age or older, and self-reported HIV-seropositivity for a minimum of 6 months prior to interview. Individuals receiving HAART must have been on treatment for at least 6 months, and also have had their HIV-1 RNA and CD4 cell counts measured within 6 months of being interviewed.

This analysis focused on the BC-sample of the national study. Separate provincial analyses were deemed necessary by the study team in light of the unique HIV epidemiological population profiles in each Canadian province. Additionally, BC had extensive pre-existing research and evidence about food security among people living with HIV, requiring further interpretation and context-specific program and policy recommendations.

Inclusion criteria for the BC dataset included: i) having completed the 24-hour dietary recall as well as the food security survey; and ii) not being pregnant in the past 12 months or currently nursing. 24-hour dietary recall data was available for a total of 904 participants from two provinces (BC and ON). After removing 592 participants, a total of 34 data outliers were removed from the sample on the basis of nutritional parameters, leaving 278 participants. Of these, 266 completed the food security survey, and 262 were neither pregnant nor nursing at the time of the survey.

There were two major community-based partners in BC: AIDS Vancouver and the Pacific AIDS Network. AIDS Vancouver (www.aidsvancouver.org) was the lead community agency for the BC component of the study. AIDS Vancouver has provided prevention, education, support, and awareness in the Lower Mainland area of BC for over 30 years. It serves an average of over 3,000 people per month with HIV through its case management program, and provides almost 18,000 bags of groceries through the Grocery Program to an average of 700 people per week. AIDS Vancouver assembled a talented multi-stakeholder team including: leaders of community-based AIDS service organizations who provided the vital community perspectives; scientists with expertise in CBR, population health and health services, food security, psychosocial and behavioural issues; leaders in the field of service delivery, advocacy, HIV policy and knowledge transfer and exchange; and HIV-positive members of the general public to share the “lived experience” of HIV. AIDS Vancouver was responsible for coordinating all community-based research activities including interview training, participant outreach, interviews, data collection and quality control. The study was also supported by the Pacific AIDS Network (PAN) (www.pacificaidsnetwork.org), a pro-active member-based coalition of over 50 member organizations working to respond to HIV, hepatitis C and related issues in BC. PAN assisted with the recruitment of community-based AIDS service organizations to participate in the study; facilitated broad-based communication and knowledge transfer of best practices in community-based research methodology across BC; and enhanced professional development of organizational members and people living with HIV through trainings and annual conferences.

The cross-sectional survey was administered in BC between March 2011 and August 2012. A provincial sampling strategy was developed, weighted to ensure adequate sample size by geographic region (i.e. Health Authority), sex and Indigenous ethnicity. Participants were recruited by PRAs using snowball sampling techniques in clinic and community settings. Snowball sampling is a method of selecting study participants from a "hidden" population. Those initially identified were asked to invite friends and acquaintances to join the study. This outreach/recruitment method was used in conjunction with the distribution of poster, flyers, newsletters and electronic information about the food security study by ASOs across BC. These approaches were used until sufficient numbers were reached to adequately power the study. Interested participants were asked to call a central toll-free telephone number for pre-study eligibility screening, and were interviewed by PRAs at participating ASO sites. All participants provided voluntary, written informed consent to participate in the study, and received a $40CAD honorarium for their time.

### Data Collection Tools

#### Quantitative survey

A quantitative survey elicited participant information about socio-demographic status, as well as behavioural, psychosocial and clinical characteristics. Where possible questions drew on validated scales in order to foster comparability of results across local and global studies. Survey questions and wording were reviewed and refined by PRAs through several iterations of focus groups discussions.

#### 24-hour dietary recall

ASA24^™^ is a freely available Web-based tool developed by the U.S. National Cancer Institute [19]. ASA24 enables automated self-administered 24-hour recalls and consists of both a respondent website used to collect recall data and a researcher website used to manage study logistics and obtain data analyses. Respondents were guided through the 24-hour recall interview using a modified version of the U.S. Department of Agriculture’s (USDA) Automated Multiple-Pass Method (AMPM) [20]. The ASA24 methodology has respondents complete the following interview steps: i) Meal-based quick list, which prompts participants to provide a list of the foods and drinks consumed at each meal occasion during the previous 24-hour recall period from midnight to midnight; ii) My Foods and Drinks list, which prompts participants if they consumed anything during any 3-hour gaps between eating occasions, as well as between midnight and the first eating occasion, and between the last eating occasion and midnight; iii) Detail Pass, prompting respondents to describe details about the foods and drinks they recorded, including form (e.g., raw), preparation methods (e.g., grilled or roasted), the amount eaten, and any additions (e.g., sugar, coffee cream, salad dressing); iv) Forgotten Foods, which probes respondents about consumption of commonly forgotten foods and drinks (e.g., snack foods, fruits, vegetables, cheese, water, coffee, tea); v) Final Review of food and drinks consumed over the 24-hour recall period, and last chance to cite any additional items consumed; and vi) Usual Intake question, which prompts respondents to respond to the question: “Was the amount of food that you ate yesterday more than usual, usual, or less than usual?” (yes vs. no). Prior to analysis, extreme outliers in ASA24 data were manually evaluated and verified in duplicate by two HIV-specialized registered dietitians (SJF and JM). Outlying variables were excluded from analysis if they exceeded the following daily thresholds: energy: 4,000 calories; carbohydrates: 700g; protein: 200g; fat: 200g; and water: 5L. These thresholds were established based on clinical experience of reasonable macronutrient intakes.

### Outcome Variable

Food insecurity was the primary dependent variable of this analysis. Adult household food insecurity was measured using Health Canada’s Household Food Security Scale Module (Canada-HFSSM), which is based on the USDA’s HFSSM [21], and has been used to assess food security across Canadian households in the Canadian Community Health Survey (CCHS) as a joint effort of Health Canada, the Public Health Agency of Canada, Statistics Canada, and the Canadian Institute for Health Information (CIHI). The Canada-HFSSM classifies the household food security status of respondents based on a group of 10 questions. Responses to each question are coded as either affirmative or negative; affirmative responses reflect any amount of food insecurity, whereas negative responses indicate food security. A dichotomous household food security variable was then constructed, characterizing respondents as food secure or food insecure based on the number of affirmative responses: two or more affirmative responses to the respective questions indicate food insecurity. Health Canada’s Household Food Security Scale Module has not been tested for validity or reliability, but assumes high specificity and sensitivity based on its similarity with USDA’s scale [22].

### Descriptive Variables

Descriptive variables selected for evaluation in this study were grouped on the basis of global definitions of food security as described by United Nations World Food Programme, Food and Agriculture Organization, the United States Agency for International Development (USAID) and salient academics [2], who understand food security as constituting: food sufficiency, dietary quality / diversity, food safety, and procurement methods.

#### Food sufficiency

A caloric intake value for the past 24 hours was generated for each participant using the ASA24. Caloric sufficiency was calculated using a modified version of the Harris Benedict Equation. The Harris Benedict Equation [23] is a method used to estimate the daily calorie requirements of an individual using their basal metabolic rate (BMR). The estimated value was then multiplied by a number that corresponded to the participant's activity level, and the final number generated was the recommended daily calorie intake to maintain current weight. There is no gold standard or recommended method for calculating daily caloric requirements for people living with HIV. However, one method for deriving daily caloric requirements for HIV-positive individuals recommended by Coyne-Meyers et al. involves generating an individual’s Harris Benedict Equation to determine daily caloric intake for weight maintenance of a non-HIV infected individual, and then multiplying it by a stress factor of 1.3 [23].

#### Dietary quality / diversity

A Healthy Eating Index–2010 (HEI–2010) score was generated to estimate dietary quality for the overall study sample using a published methodology [24]. The HEI score measures intake of ten dietary components, providing a single score out of a total 100 points. Diets with an HEI score >80 are interpreted as “good,” >51- ≤80 as “fair,” and <51 as “poor” [25, 26]. The HEI–2010 has been specifically designed to assess diet quality of a population or subgroups defined by income, race/ethnicity, and other characteristics; and to examine relationships between overall diet quality and outcomes, such as mortality or incidence of some chronic diseases [27]. The HEI–2010 differs from its previous iteration, the 2005 version, in several respects, including the fact that it reflects changes in the 2010 Dietary Guidelines for Americans, and places emphasis on impact of seafood, plant proteins and refined grains [28]. Basic steps for calculating HEI-2010 include:

1. identifying a set of foods under consideration (those listed by participants in 24-hr recall)
2. determining the amount of each relevant dietary constituent in the set of food groups (total fruit; whole fruit [excluding juice]; total vegetables; beans and peas; dark green vegetables; whole grains; dairy [milk, yogurt, cheese, and fortified soy beverages in the form of skim milk equivalents]; total protein foods [lean fraction only]; seafood; nuts and seeds; refined grains; saturated fatty acids; polyunsaturated fatty acids; monounsaturated fatty acids; sodium; calories from added sugars, solid fats, and alcohol [separately]; and total calories
3. deriving pertinent ratios (i.e. density values) and score of each HEI component using the relevant standard.

Data collected at the individual level through the 24-hour recall tool were coded using either of the USDA's Food and Nutrient Database for Dietary Studies (FNDDS). These databases provide compositional information for a full array of nutrients. The FNDDS links to the MyPyramid Equivalents Database, which characterizes the foods reported according to components needed to calculate the HEI. The majority of values generated by the HEI score are comparable to the Canadian Healthy Eating Guideline Recommended Daily Intake (RDI).

#### Food safety

Food safety at the household level was measured using the following questions: current access to a refrigerator (yes vs. no); hand washing before preparing food (all/most of the time vs. sometimes/rarely); within past six months (yes vs. no); reported sickness from spoiled/unsafe food within past six months (yes vs. no); physician-confirmed food-borne sickness within past six months (yes vs. no).

#### Food procurement methods

The following individual variables were used to investigate potential methods of food procurement in the past 12 months: attendance at any food distribution, bank or program (yes vs. no); attendance at food program specific for people living with HIV (yes vs. no); begged or panhandled for food (yes vs. no); stole food (yes vs. no); traded sex for food (yes vs. no); traded drugs for food (yes vs. no); sold/pawned for food (yes vs. no); borrowed money to purchase food (yes vs. no); and dumpster dove (yes vs. no). An additional dichotomous variable was constructed to capture *any* procurement of food using non-traditional methods in the past 12 months by collapsing all affirmative responses to the above questions into a single category (excluding only the question regarding food distribution, bank or program).

#### Socio-demographic and behavioural

Socio-demographic and behavioural variables investigated included: age (median); biological sex (male vs. female); current location of residence (urban vs. rural), derived postal code structure based on Canada Post guidelines [29] and is consistent with Statistics Canada definitions of rural areas [30]; Indigenous ancestry (yes *vs*. no); sexual orientation (gay/lesbian/bisexual/two-spirited vs. straight); employed in past 30 days (yes vs. no); currently receiving disability insurance (yes vs. no); highest education ever completed (no formal education/some elementary school/completed elementary school/some high school /completed secondary school vs. other); household gross yearly income (<$20,000 vs. ≥$20,000) [dichotomous split approximates Statistics Canada low income cut-off for 2009 [31]]; current unstable housing (self-contained room in a motel, hotel or boarding house/housing facility/outdoors/couchsurfing/shelter vs. other).

Behavioural variables included: tobacco smoking in the last 30 days (yes vs. no); cannabis use in the last 30 days (yes vs. no); any illicit drug use in the past 12 months (yes vs. no); and alcohol dependency (yes vs. no) assessed with the 10-item Alcohol Use Disorders Identification Test (AUDIT) [32]. AUDIT was designed to identify individuals whose alcohol use places them at risk for the development of an alcohol use disorder. Each participant’s score was obtained by adding the 10 items for a total score ranging from zero to 40. Participants were characterized as either “abstainers” (AUDIT = 0), “nonhazardous drinkers” (AUDIT score = 1–7) or “hazardous drinkers” (AUDIT ≥ 8). Depressive symptoms in past six months were assessed using the 10-item Center for Epidemiological Studies Depression scale (CES-D 10). Participants with scores of 10 or higher were identified as having depressive symptoms. This scale has been shown to have good predictive accuracy when compared to the 20-item scale (kappa = 0.97, p <0.0001) [33]. Social support was measured using the Medical Outcomes Study (MOS) Social Support Survey Score [34]. HIV stigma was measured using a modified version of the HIV Stigma scale, which has shown to be reliable and valid within a diverse sample of people living with HIV [35].

### Statistical Analysis

Variable selection and analytic techniques employed in this study built on a body of existing literature focused on food security among people living with HIV in this setting. All analyses were conducted using SAS version 9.1.3 (SAS Institute, Cary, North Carolina, U.S.A.). We conducted bivariate analysis to ascertain variables associated with food insecurity among people living with HIV. Chi-square tests were used to compare categorical variables. In instances where expected counts were small (five or less), Fisher’s exact tests were used. Continuous variables were compared using Wilcoxon rank sum test. All tests of significance were two-sided, with a cut-off p-value of <0.05.

In multivariable explanatory modeling, a backward-selection procedure based on the Akaike Information Criterion and Type III p-values were used to select variables for inclusion in the final model [36] and the Concordance Index was used to determine final model fit [37]. Tolerance and Variance Inflation Factor values were calculated to assess possible multicollinearity of explanatory variables in the final model [38]. All tests of significance were two-sided, with a *p*-value less than 0.05, or 95% CI not overlapping 1.0, indicating a statistically significant association.

### Ethics Statement

All study procedures underwent research ethics board (REB) approval at the following participating institutions: Providence Health Care (PHC) / University of British Columbia (UBC) [#H09-02494]; Simon Fraser University (SFU) [#2010s0524]; University of Toronto [#25710]; and McGill University Health Centre (MUHC) [#02-065GEN]. All participants provided voluntary, written informed consent to participate in the study.

## Results

Between March 2011 and August 2012, 266 participants completed both the quantitative survey and the 24-hour dietary recall. After removing participants who reported being pregnant or nursing, data from 262 participants were deemed eligible for analysis (see S1 Dataset). A total of 192 (73%) participants reported being food insecure. The median age of participants was 47 years (inter-quartile range [IQR]: 41–51), 191 (73%) were male, and 91 (35%) reported Indigenous ancestry.

Table 1 describes proportional values for responses to individual questions within the Canada—HFSSM. Questions with the highest proportion of participant responses included those alluding to feelings of anxiety regarding food sufficiency (70%), food running out (70%), and not being able to afford balanced meals (72%). Table 2 describes participant socio-demographic and behavioural characteristics by food security status. Factors significantly associated with food insecurity included not being employed in the past 30 days, household gross yearly income <$20,000, unstable housing, cigarette smoking in the past month, illicit drug use in the past 12 months, HIV stigma and symptoms of depression (*p* <0.05). Table 3 describes bivariate comparison of daily participant caloric and nutrient sufficiency by food security status. Of the energy and nutrient intake variables examined, ‘below DRI of protein’ was the only variable associated with food insecurity (p = 0.046). Table 4 describes HEI scores for the entire study sample. The overall HEI score for the study sample was 57.04 out of a total possible score of 100, indicating a “fair” quality of diet. HEI components that scored low compared to total possible score for each category included: seafood and plant protein (2.53/5), fatty acids (3.32/10) and sodium (3.13/10), dairy (5.97/10), and empty calories (10.97/20). Food secure and insecure individuals differed significantly in their overall HEI score (p = 0.01), total fruit (without juices) (p = 0.035), and protein (p = 0.033). Table 5 describes results for bivariate analyses of food safety characteristics associated with food insecurity. Of the food safety variables examined, “being sick from spoiled/unsafe food in the past six months” was the only variable associated with food insecurity (*p =* 0.010). Table 6 presents findings from the bivariate comparison of food procurement methods used in the past 12 months, by food security status. All variables examined were found to be associated with food insecurity (p <0.05); of these, having “attended any food distribution/bank/program”, “sold/pawned for” and “stolen” food in the past 12 months were significantly associated with food insecurity (*p* <0.001). Table 7 presents univariate and multivariate analyses of factors associated with food insecurity. After controlling for potential confounders, factors significantly associated with food insecurity included: non-traditional methods of food procurement [adjusted odds ratio (AOR) = 11.11, 95% confidence interval (CI): 4.79–25.68, p = <0.001]; younger age [AOR = 0.92, 95% CI: 0.86–0.96, p = <0.001]; unstable housing [AOR = 4.46, 95% CI: 1.15–17.36, p = 0.031]; household gross annual income [AOR = 4.49, 95% CI: 1.74–11.60, p = 0.002]; and symptoms of depression [AOR = 2.73, 95% CI: 1.25–5.96, p = 0.012]. The goodness of fit for the final model was assessed by the Concordance Index (c = 0.845), which suggested no indication of lack of fit. The variance inflation factor (VIF) was examined for each variable, all of which were less than 1.05, respectively. These values indicate that there were no concerns with multi-co-linearity of explanatory variables in the final model.

**Table 1:** Participant responses to Canada Household Food Security Scale Module.

	Overall n = 262	
	n	(%)
**In the past 12 months you and other household members worried that food would run out before you got money to buy more**		
Never true	79	(30)
Sometimes/Often true	183	(70)
**In the past 12 months the food that you and other household members bought just did not last and there was not any money to get more**		
Never true	79	(30)
Sometimes/Often true	183	(70)
**In the past 12 months the food that you and other household members bought just did not last and there was not any money to get more**		
Never true	79	(30)
Sometimes/Often true	183	(70)
**In the past 12 months you and other household members could not afford to eat balanced meals**		
Never true	73	(28)
Sometimes/Often true	189	(72)
**In the past 12 months since the last [current month] did you or other adults in your household ever cut the size of your meals or skip meals because there was not enough money for food?**		
No	115	(44)
Yes	147	(56)
**How often did this happen?**		
Almost every month	76	(29)
Some months but not every month	43	(16)
Only one or two months	28	(11)
**In the past 12 months did you ever eat less than you felt you should because there was not enough money to buy food?**		
No	116	(44)
Yes	146	(56)
**In the past 12 months were you ever hungry but did not eat because you could not afford enough food?**		
No	128	(49)
Yes	134	(51)
**In the past 12 months did you lose weight because you did not have enough money for food?**		
No	153	(58)
Yes	109	(42)
**In the past 12 months did you or other adults in your household ever not eat for a whole day because there was not enough money for food?**		
No	185	(71)
Yes	77	(29)
**How often did this happen?**		
Almost every month	27	(10)
Some months but not every month	27	(10)
Only one or two months	23	(9)

**Table 2:** Bivariate comparison of participant socio-demographic and behavioural characteristics, by food security status (n = 262).

	Food Secure		Food Secure		
Characteristic	No		Yes		P-value
	n (%)		n (%)		
	median (Q1-Q3)		median (Q1-Q3)		
	192 (73%)		70 (27%)		
**Median age**	46	(41–50)	49	(44–57)	<0.001
**Sex at birth**					
Male	140	(73)	51	(27)	0.992
Female	52	(73)	19	(27)	
**Indigenous ethnicity**					
No	119	(71)	49	(29)	0.241
Yes	71	(78)	20	(22)	
**Sexual orientation**					
Heterosexual/Straight	123	(75)	41	(25)	0.471
Gay/Bisexual/Two-spirited/Other	69	(70)	29	(30)	
**Highest education level completed**					
High school or less	122	(76)	39	(24)	0.250
More than high school	68	(69)	31	(31)	
**Residence**					
Urban	134	(70)	58	(30)	0.556
Rural	3	(100)	0	(0)	
**Employed, past 30 days**					
No	167	(76)	53	(24)	0.033
Yes	24	(59)	17	(41)	
**Household gross yearly income**					
< $20,000	156	(78)	43	(22)	<0.001
≥$20,000	15	(42)	21	(58)	
**Unstable housing**					
Room in a motel/hotel/boarding house/ facility; couch surfing; shelter	41	(87)	6	(13)	0.016
Other	137	(70)	59	(30)	
**Smoked cigarettes, past one month**					
No	39	(57)	29	(43)	0.001
Yes	153	(79)	41	(21)	
**Alcohol dependent (AUDIT score)**					
No	69	(72)	27	(28)	0.695
Yes	39	(76)	12	(24)	
**Illicit drug use, past 12 months**					
No	59	(63)	35	(37)	0.005
Yes	126	(79)	33	(21)	
**HIV stigma**					
No	85	(63)	50	(37)	<0.001
Yes	107	(84)	20	(16)	
**Social support (MOS scale)**					
Less support	91	(70)	39	(30)	0.265
More support	101	(77)	31	(23)	
**Depressive symptoms**					
No	75	(64)	43	(36)	0.001
Yes	116	(82)	26	(18)	

**Table 3:** Bivariate comparison of daily participant caloric and nutrient sufficiency by food security status (n = 262).

	Food Secure		Food Secure		P-value
Characteristic	No		Yes		
	n (%)		n (%)		
	192 (73%)		70 (27%)		
**Caloric sufficiency**					
**Energy intake (Kcal)**					
Median (IQR)	1840	(1293–2578)	1796	(1364–2256)	
**HIV-specific energy sufficiency (Kcal)** *					
No	50	(78)	14	(22)	0.314
Yes	142	(72)	56	(28)	
**Dietary Diversity**					
**Nutrient intake (g) by DRI** †					
Vegetable					
Below	179	(74)	63	(26)	0.384
At or above	13	(65)	7	(35)	
Fruit					
Below	132	(73)	48	(27)	0.978
At or above	60	(73)	22	(27)	
Protein					
Below	78	(80)	19	(20)	0.046
At or above	114	(69)	51	(31)	
Fat					
Below	35	(81)	8	(19)	0.188
At or above	157	(72)	62	(28)	
Carbohydrate					
Below	39	(78)	11	(22)	0.402
At or above	153	(72)	59	(28)	

* Notes: Derived using Harris Benedict Equation.

^†^ Notes: Daily Recommended Intake (DRI), based on Canada Healthy Eating Food Guide: fruits and vegetables (7–8 servings for men; 8–10 servings for women); protein (34–56 for men / 34–46 for men) based on age; fat (30g); carbohydrate (130g).

**Table 4:** Bivariate comparison of participant Health Eating Index (HEI) by food security status.

Component	Food Insecure	Food Secure	*P-value*
	*N = 192*	*N = 70*	
Total vegetables			
Median	3.1	4.15	0.066
25th- 75th percentile	(1.57–4.85)	(1.72–5)	
Greens & beans			
Median	0	0	0.949
25th- 75th percentile	(0–2.7)	(0–2.76)	
Total fruits			
Median	3.8	3.66	0.441
25th- 75th percentile	(0.54–5)	(1.9–5)	
Fruit (without juices)			
Median	0.41	2.3	0.035
25th- 75th percentile	(0–5)	(0–5)	
Whole grains			
Median	1.82	1.2	0.543
25th- 75th percentile	(0–6.22)	(0–4.28)	
Dairy			
Median	4.92	4.91	0.756
25th- 75th percentile	(1.45–8.7)	(2.47–8.27)	
Total protein			
Median	4.23	5	0.033
25th- 75th percentile	(1.97–5)	(3.23–5)	
Seafood & plant protein			
Median	0.03	0.04	0.476
25th- 75th percentile	(0–1.51)	(0–4.8)	
Fatty acids			
Median	3.8	2.98	0.923
25th- 75th percentile	(0–7.74)	(0.86–8.12)	
Sodium			
Median	3.64	3.94	0.385
25th- 75th percentile	(0–7.37)	(0.25–9.22)	
Refined grains			
Median	6.88	8.92	0.085
25th- 75th percentile	(3.32–10)	(4.32–10)	
Empty calories			
Median	11.38	12.96	0.077
25th- 75th percentile	(5.79–16.89)	(10.25–17.05)	
Overall			
Median	47.41	51.67	0.01
25th- 75th percentile	(38.92–55.63)	(43.09–62.64)	

**Table 5:** Bivariate comparison of participant food safety by food security status (n = 262).

	Food Secure		Food Secure		P-value
Characteristic	No		Yes		
	n (%)		n (%)		
	192 (73%)		70 (27%)		
**Access to a refrigerator**					
No	8	(100)	0	(0)	0.113
Yes	170	(72)	65	(28)	
**Hand washing before food preparation**					
All/most of the time	165	(72)	65	(28)	0.367
Sometimes/rarely	23	(82)	5	(18)	
**Sick from spoiled/unsafe food, past six months**					
No	140	(69)	63	(31)	0.009
Yes	46	(87)	7	(13)	
**Physician confirmed food-borne sickness, past six months**					
No	9	(82)	2	(18)	0.479
Yes	9	(100)	0	(0)	

**Table 6:** Bivariate comparison of procurement methods by food security status (n = 262).

	Food Secure		Food Secure		P-value
Characteristic	No		Yes		
	n (%)		n (%)		
	192 (73%)		70 (27%)		
Any non-traditional methods of food procurement*					
No	53	(50)	53	(50)	<0.001
Yes	138	(89)	17	(11)	
**Attended food distribution/bank/program**					
No	17	(47)	19	(53)	<0.001
Yes	174	(77)	51	(23)	
**Beg or panhandled for food**					
No	167	(71)	68	(29)	0.019
Yes	24	(92)	2	(8)	
**Stole food**					
No	156	(70)	68	(30)	0.002
Yes	35	(95)	2	(5)	
**Traded sex for food**					
No	180	(72)	70	(28)	0.040
Yes	11	(100)	0	(0)	
**Traded drugs for food**					
No	174	(72)	69	(28)	0.049
Yes	17	(94)	1	(6)	
**Sold/pawned for food**					
No	140	(67)	69	(33)	<0.001
Yes	50	(98)	1	(2)	
**Borrowed money for food**					
No	66	(54)	56	(46)	<0.001
Yes	124	(90)	14	(10)	
**Procured dumpster or discarded food**					
No	165	(71)	69	(29)	0.002
Yes	26	(96)	1	(4)	

* This composite variable includes all methods of procurement listed, except ‘attended any food distribution/bank/program’.

**Table 7:** Univariate and multivariate analyses of factors associated with food insecurity (n = 218).*

	Univariate Model		Multivariate Model	
	OR (95% CI)	p-value	AOR (95% CI)	p-value
**Any non-traditional food procurement**				
No vs. yes	10.11 (4.90, 20.84)	<0.001	11.11 (4.79, 25.68)	<0.001
**Age**				
Per 10 year increase	0.92 (0.89, 0.96)	0.001	0.91 (0.86, 0.96)	<0.001
**Unstable housing**				
No vs. yes	5.13 (1.51,17.41)	0.009	4.46 (1.15,17.36)	0.031
**Household gross annual income**				
< $20,000 vs. ≥$20,000	5.49 (2.54, 11.86)	<0.001	4.49 (1.74,11.60)	0.002
**HIV stigma**				
No vs. yes	2.65 (1.4, 5.01)	0.003		
**Depressive symptoms**				
No vs. yes	2.54 (1.37, 4.7)	0.003	2.73 (1.25, 5.96)	0.012

* For supporting information see S1 Dataset.

## Discussion

This study evaluated the relationship between food insecurity and its sub-components in a community-based sample of people living with HIV across the province of BC, Canada. Seventy-three percent of participants in this study reported being food insecure. This prevalence value was identical to that reported by a separate cohort of HIV-positive individuals in BC two years earlier using a different food security measurement tool [3], and highlights both the reliability of our findings and the severity of food insecurity in this population. The proportion of food insecure people living with HIV in BC is nine times higher than the general population in the province [39] and 20% higher than food insecurity levels reported among HIV-positive homeless and marginally housed individuals in San Francisco, measured using a similar food security scale [8]. Socio-demographic and clinical factors associated with food insecurity included: younger age, unstable housing and symptoms of depression. Notably, study participants who were food insecure had over 10 times increased odds of reporting non-traditional procurement of food.

Implicit in the concept of food insecurity is that a person living with HIV who is food insecure may be accessing or utilizing limited quantity, quality or safety of foods, or procuring them using ‘socially unacceptable’ or non-traditional manners [2]. In an effort to better direct evidence-based programs and policies, we sought to delineate the primary components of food insecurity experienced by this population. Bivariate comparison of daily participant caloric and nutrient sufficiency found that participants who reported being food insecure did not differ in their energy (caloric) intake compared to those who were food secure. However, food insecure individuals were more likely to report poorer dietary quality. With an overall sample HEI median score of 57, we may venture to conclude that the majority needed improvement in their diets and that many would be classified as having a poor quality diet. The group HEI score suggested that participants consumed a significant number of ‘empty calories’, moderate amounts of seafood and plant protein, and limited dairy. We found that food secure and insecure individuals differed significantly in their overall HEI score (p = 0.01), total fruit (without juices) (p = 0.035), and protein (p = 0.033). These findings suggest that people living with HIV who are food insecure require additional social supports to ensure improved access to diverse diets that are richer in whole fruits and protein sources.

Taken together with findings that 77% of food insecure individuals in this cohort were accessing charitable food distributions at time of interview, there is a clear need for improved dietary quality at food distribution points accessed by people living with HIV across BC. Addressing the dietary quality of foods consumed by this population is important in light of evidence regarding the nutritional pathways linking food insecurity to adverse HIV clinical outcomes [13], and notably the role of specific micronutrient deficiencies, such as selenium and zinc, in HIV-related disease progression and risk of death [40–45].

We found that being sick from spoiled/unsafe food was strongly associated with food insecurity in bivariate analysis. These findings support research that suggests people living with HIV have increased social and biological risk of acquiring foodborne pathogens. Few epidemiologic studies have evaluated the incidence of foodborne diseases among people living with HIV. However a study conducted early in the HIV epidemic estimated that salmonellosis was approximately 20 times more common in AIDS patients than those without AIDS [46]. A more recent study in South Africa found 48 of 60 HIV-positive patients with chronic diarrhea had bacterial pathogens [47]. While the association between food-borne illness symptoms and food insecurity did not persist in multivariable analysis, our findings highlight the importance of monitoring and the possible role of food safety as an important sub-component in the experience of food insecurity in this population.

Although public health authorities and researchers in North America recognize food procurement methods as a defining aspect food security [48, 49], this is the first study to our knowledge to have comprehensively assessed food procurement methods among people living with HIV. In bivariate analysis, we found that participants who procured food using non-traditional methods—namely reported having begged or panhandled for food, having stolen food, traded sex for food, traded drugs for food, sold/pawned for food, borrowed money for food and dumpster-dived for food—were all more likely to be food insecure. The most salient finding from this study is the fact that, when examined as a composite indicator, study participants who were food insecure had 10 times increased odds of reporting non-traditional procurement of food. These findings highlight the urgent need to address the social context of food insecurity among people living with HIV in BC. Several studies have found that food insecurity is associated with HIV-related stigma [50, 51], both enacted (where an individual perceives adverse acts directed them due to their HIV status) and internalized (where an individual appropriates a disempowered or undervalued self-perception due to their HIV status) [52]. Food insecurity has shown to lead to increased HIV disease progression through behavioural mechanisms by contributing to ART non-adherence, missed clinical visits and treatment interruptions [13]. Our study results suggest that addressing social and behavioural aspects of the experience of food insecurity is essential to ensuring the health and wellbeing of people living with HIV in this setting.

We found that participants reporting low socio-economic status and unstable housing had 4.5 times increased odds of being food insecure. This finding echoes results from a study carried out in separate cohorts of people living with HIV in this setting [3, 11], and adds to a growing body of literature that shows that housing is an important determinant of people living with HIV’s overall wellbeing. Studies evaluating food insecurity among non-HIV infected North Americans have similarly reported associations between poor nutritional status and low income and education levels [53–56]. Follow-up studies should evaluate the extent to which this population has access to social support services, and whether the services have any positive mitigating impact on food insecurity.

Consistent with findings from a study we have previously carried out among people living with HIV in this setting, individuals reporting symptoms of depression had 2.7 times increased odds of being food insecure [3]. The association between food insecurity and depression has been delineated within non-HIV-infected populations [57–60]. Food insufficiency has been associated with poor mental health status among adults and adolescents, including symptoms of depression, dysthymia and suicide [57, 59]. Conversely, depressive symptoms have been associated with insufficient food intake, and reduced consumption of quality foods, particularly among women [61, 62]. The prevalence of major depressive episodes or generalized anxiety disorders may increase with food insecurity [60]. Among people living with HIV, depression is hypothesized to be a pathway through which food insecurity leads to adverse HIV clinical outcomes, and may be bidirectional [13]. The finding that symptoms of depression are associated with food insecurity is consistent with studies in San Francisco that found food insecurity among HIV-infected homeless and marginally housed individuals was strongly associated with poor mental health status [4, 63]. It also supports a study that found significant decreases in dietary macronutrient intake among people living with HIV who developed depression, compared to those who did not [64]. Future research is required to determine whether depression is a cause or consequence of food insecurity, and to ascertain the extent to which depression aggravates food insecurity among individuals on ART. In the meantime, nutrition support services aimed at HIV-infected individuals in BC should explore programmatic links with mental health services.

Our study has specific implications for health planners and policymakers. First, while the prevalence of food insecurity is very high in this sample, it is important to note that the modest financial incentive given to participants may have increased the probability of sampling people who cannot afford sufficient, safe and nutritious food. Social support services for people living with HIV should consider the individual health and nutritional needs on case-by-case basis to optimally support clients. Second, although the vast majority of food insecurity individuals in this sample (77%) reported having access to some type of food distribution, bank or program, our findings suggest that significant improvements need to be made to the quality and diversity of foods access by people living with HIV. Third, the extremely high proportion of food insecure individuals reporting non-traditional food procurement methods (89%) suggests an urgent need for outreach services within food support programs to optimize people’s access to food. Where possible, food supplementation programs may benefit from aligning outreach initiatives with existing HIV, sexual health and harm reduction programs aimed at ‘hard to reach populations’.

### Strengths and Limitations

This study has several limitations. Due to the cross-sectional design, this study cannot infer causality, or potential social, structural and biologic mechanisms that may link food insecurity to characteristics. Study findings are not generalizable to the entire population of people living with HIV in BC or other provinces because participant sampling was not randomized. The food security scale used in this analysis has been shown to have high sensitivity and specificity, strengthening research confidence in the accuracy of point estimates for food security status. The ASA24 tool used in this study was originally developed for use and analytic comparison with U.S. population parameters and U.S. dietary guidelines. Accordingly, results regarding adequacy of dietary intake do not reflect important differences with Canadian dietary guidelines. Indeed, an evaluation of the National Cancer Institute’s Diet History Questionnaire and nutrient database used in this study found that errors in nutrient intake estimates may exist due to differences between American and Canadian food fortification laws and practices, leading to possible overestimation or underestimation of nutrient intake in Canadian population samples depending on food items consumed [65]. As a retrospective dietary intake assessment tool, 24-hour recalls are subject to recall bias. However, the methodology overcomes many barriers for vulnerable populations such as literacy and numeracy when compared to similar measurement techniques. The modest financial incentive given to participants may have increased the probability of sampling people who are food insecure, thereby inflating prevalence estimates and limiting generalizability. Information (measurement) bias, and specifically non-differential misclassification of food insecurity status may have led food insecure participants to be equally misclassified among exposed and non-exposed groups, biasing adjusted measures of association towards the null. Accordingly, it is possible that the strengths of association between food insecurity and explanatory variables were underestimated. Because survey data were self-reported, this study may have also been susceptible to recall bias and social desirability bias. However, there is no reason to believe that the magnitude of these biases would differ between food secure and insecure groups. This study did not use anthropometrics or biomarkers to evaluate nutritional status, which may have allowed for more rigorous evaluation of nutritional status. A strength of the logistic regression technique used is that it prioritized elimination of bias over parsimony. In our model building, we intentionally excluded the use of automated selection procedures, and employed a conservative manual backward selection technique, which reduced risk of bias from residual confounding

## Conclusion

We found that food insecurity is highly prevalent in our sample of people living with HIV and characterized by consumption of diets that are poor in dietary quality and procured in non-traditional manners. Our study provides essential data to guide evidence-based local programs and policies aimed at preventing and mitigating food insecurity among people living with HIV. Future operational research is needed to assess the effectiveness of structural interventions aimed at strengthening dietary quality and social access to food in this setting.

## Supporting Information

S1 Dataset (XLS)
